# Exploring different methods of cellulose extraction for ^14^C dating

**DOI:** 10.1039/d1nj00290b

**Published:** 2021-04-29

**Authors:** Silvia Cercatillo, Michael Friedrich, Bernd Kromer, Dragana Paleček, Sahra Talamo

**Affiliations:** Department of Chemistry G. Ciamician, BRAVHO Radiocarbon Laboratory, Alma Mater Studiorum, University of Bologna, Via Selmi 2 Bologna 40126 Italy; Hohenheim Gardens, University of Hohenheim, Emil-Wolff-Strasse 38 Stuttgart D-70599 Germany; Institute of Environmental Physics, Heidelberg University Heidelberg D-69120 Germany

## Abstract

In this study we aim to identify the optimal cellulose extraction protocol for ^14^C dating of wood, with a focus on glacial trees. To achieve this, we compare three cellulose extraction methods on the basis of cellulose yield and ^14^C age. The study is conducted on 12 wood samples of different species, in varying states of preservation with ages covering the full ^14^C age range. Cellulose is extracted from each sample following three different protocols selected from the literature: ABA-B, BABAB and 2Chlorox. The extracted cellulose was graphitised and dated with the MICADAS (Mini Carbon Dating System) at the ETH AMS laboratory. Although all three methods are considered efficient, the BABAB protocol, despite being a more aggressive procedure, allows the extraction of a sufficient amount of cellulose to be ^14^C dated and leads to the most reliable results, particularly for very old and background samples (samples with ^14^C content of zero).

## Abstract 2

Evaluation of different cellulose extraction protocols to obtain reliable radiocarbon ages especially from trees of glacial period.

## Graphical abstract

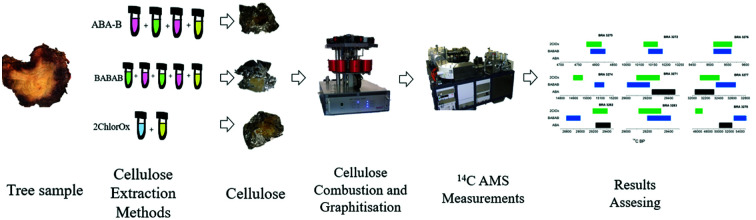

## Introduction

Cellulose (C_6_H_10_O_5_) is an abundant biopolymer. The use of this compound is spreading in many fields, including the biomedical, packaging and environmental sectors, thanks not only to its chemical and physical properties, but also as a renewable, sustainable and biodegradable material.^4–6^ In this study we chemically isolate cellulose from wood to exploit another of its unique qualities: its record of atmospheric carbon content.^7,8^ Through the photosynthesis process, atmospheric CO_2_ is stored as carbon in plants. During growth, the ratio of carbon isotopes in the plant tissues is in equilibrium with the atmosphere. After active growth has ceased, the radioactive carbon isotope (^14^C) concentration starts to decrease. The concentration of ^14^C can be measured with annual resolution from the cellulose extracted from tree rings.^8^ Radiocarbon is the most precise and universal tool for dating events of the last 55 000 years,^9^ the limit of the radiocarbon method. Here we study sub-fossil tree remnants (wood fragments in which the organic component is not fully decomposed and is only partially fossilized) grown in the glacial period. We intend to improve the resolution of the radiocarbon calibration curve (used to convert ^14^C ages into calendar ages) beyond the current extent of the unbroken dendrochronological curve (back to 14 226 years cal BP††Conventional term for radiocarbon calibrated dates corresponding to calendar years considering 1950 as present. Cal – (calibrated) BP – before present (1950).) to allow more precise age determinations.^9^ Cellulose is the fraction of choice for dating trees.^2,7,8,10^ To obtain reliable ^14^C ages any contamination by extraneous carbon in the sub-fossil wood must be removed. Contamination can occur by young carbon, *e.g.* humic acid introduced from groundwater supply, or from ^14^C-free carbonates. For radiocarbon dating of Upper Pleistocene samples, contamination by young carbon is more problematic than contamination by fossil carbon: 1% of modern carbon contamination added to a sample of 37 000 ^14^C BP will reduce its age by 5500 years, whereas 1% ^14^C-free carbon will increase the age by only 80 years. Therefore, when assessing the effectiveness of pretreatment procedures, older ages are generally considered more reliable. Several studies report cellulose extraction protocols for young Holocene trees as well as wood near/beyond the limit of ^14^C dating (>55 000 years BP).^1,3,11^ We want to identify the most effective pretreatment procedure to extract cellulose and efficiently remove exogenous contamination from sub-fossil trees spanning the glacial period. We compare three different protocols, including a novel technique which has been recently published. We selected subfossil trees of different species from different time periods to identify the limits and capabilities of each extraction method. For most of the samples the age is already known, either from dendrochronology or from radiocarbon dating. The results are evaluated on the basis of the cellulose yield and the ^14^C age.

## Experimental

### Materials and methods

Twelve wood samples within the age range of ^14^C dating were selected from our archive (Table 1). Samples (*Pinus sylvestris* L.) from Furadouro were found in an ancient forest bed under the sandy shore of the northern Atlantic coast of Portugal, near the city of Ovar, accessible only during rare events of minimum low tide.^12^ In this study, three sub-samples of the same tree section were dated. The sample from Revine (*Larix decidua*) was found in a larch forest preserved in colluvial and lacustrine deposits near the lake of Revine (Treviso) in the Italian Pre-Alps.^13^ Cairo Montenotte (*Pinus sylvestris* L.) is part of a sub-fossil pine tree stump sampled from glacial sediments in Savona (Italy) in 2018. The conifer wood sample from Reichwalde is a background sample,^14^ which was found in Miocene layers in the lignite mining area in Saxony, Germany.^15^ Wood samples from Győrújfalu (*Fraxinus spec.*) and Győrzámoly (*Ulmus spec.*) were found in Hungary, near the city of Győr, in Quaternary gravel sediments in a tributary of the Danube River. The Győrzámoly sample was previously dated at the Heidelberg radiocarbon laboratory to beyond the ^14^C dating limit (>55 000 years BP). Recently, samples from Győrújfalu were unsuccessfully pretreated (cellulose was not obtained) at Mannheim radiocarbon laboratory. To verify their preservation state, they were tested again. The sample from Illegio is a larch stump (*Larix decidua*) found in sediments dating to the LGM (Last Glacial Maximum) in the basin of the Tagliamento River in the Carnic alps of the Friuli-Venezia-Giulia region (Italy), as described in Monegato *et al.*^16^ The sample analysed here was found *in situ* together with another larch trunk, dated by Hajdas *et al.*^17^ The Late Glacial pine (*Pinus sylvestris* L.) from Breitenthal was found in a gravel pit in southern Germany near the Günz River, together with other pines. They were dendrochronologically analysed by Friedrich *et al.*^18^ Samples from Ebensfeld and Freising are Holocene oak (*Quercus spec.*) and pine (*Pinus sylvestris* L.) found in Quaternary deposits of the rivers Isar and Main in southern Germany. They were remnants of old riparian forests buried by river activity and found in gravel pits. They are parts of the Holocene oak and pine chronologies of central Europe.^18,19^

**Table 1:** Sample information. Samples are ordered by age (from youngest to oldest)

Bologna lab code BRA-	Site	Species	Ref.	Dendro-date or ^14^C age (if floating)
3275	Ebensfeld, Germany	Oak (*Quercus spec.*)	Friedrich *et al.*;^19^ Friedrich *et al.*^18^	Ring 60–85 (72): 5569 BP (3620 BC)
3276	Freising, Germany	Pine (*Pinus sylvestris* L.)	Friedrich *et al.*;^19^ Friedrich *et al.*^18^	Ring 20–40 (30): 10.872 BP (8923 BC)
3272	Breitenthal, Germany	Pine (*Pinus sylvestris* L.)	Friedrich *et al.*^19^	Ring 50–80 (65): 11.840 BP (9891 BC)
3274	Revine, Italy	Larch (*Larix decidua*)	Kromer *et al.*;^20^ Friedrich *et al.*;^19^ Kaiser *et al.*^21^	∼15 000 ^14^C BP
3271	Furadouro, Portugal	Pine (*Pinus sylvestris* L.)	This study	∼27 000 ^14^C BP (untreated)
3282	Furadouro, Portugal	Pine (*Pinus sylvestris* L.)	This study	∼27 000 ^14^C BP (untreated)
3283	Furadouro, Portugal	Pine (*Pinus sylvestris* L.)	This study	∼27 000 ^14^C BP (untreated)
3277	Illegio, Italy	Larch (*Larix decidua*)	This study	Not dated
3278	Cairo Montenotte, Italy	Pine (*Pinus sylvestris* L.)	This study	Not dated
3279	Győrújfalu, Hungary	Ash (*Fraxinus spec.*)	This study	Background (probably Eemian)
3281	Győrzámoly, Hungary	Elm (*Ulmus spec.*)	This study	Background (probably Eemian)
3273	Reichwalde, Germany	Conifer (*c.f.* Chamaecyparis)	Sookdeo *et al.*;^14^ Scott *et al.*^22^	Background (Miocene)

### Pretreatment methods

Three sub-samples were taken from each of the 12 trees, one for each protocol. Nine sub-samples weighed ∼200 mg, and 3 samples ∼40 mg to ensure that all methods could be applied in cases of limited material. All samples were chopped into small pieces and were put into 10 mL glass tubes and processed according to the three different protocols: ABA-B,^1^ BABAB^2^ and 2ChlorOx.^3^ The classic ABA method^1,8,23^ was tested with the addition of a final bleaching step (ABA-B).^1^ The BABAB method adds an initial overnight bath in alkaline solution to the ABA method followed by a final oxidative bleaching step.^2^ The recently published 2ChlorOx method involves an alkaline hypochlorite and an acidic chlorite oxidation bleaching step, repeated two times.^3^

#### ABA-B^1^

Samples were treated with 4% HCl to remove contamination from carbonates, then washed with MilliQ water, followed by a 4% NaOH bath, and a second 4% HCl step to remove any absorbed atmospheric CO_2_. Each step was carried out for ∼1 hour. A bleaching solution (5% NaClO_2_ and some drops of 4% HCl) to remove lignin and other contaminants was applied for ∼1 hour,^24,25^ then renewed to increase efficiency, as suggested by Capano *et al.*,^1^ and left for another hour. All procedures are carried out in a heater-block at 75 °C. The final extract was dried in an oven at 80 °C.

#### BABAB^2^

Samples were left in a 4% NaOH bath at 75 °C overnight to dissolve the lignin (making the cellulose more accessible to reagents)^2^ and the humic acid (soluble at high pH) from the soil.^26,27^ The following day, the acid–base–acid steps (as described above for the ABA-B protocol) were performed. The final bleaching step to remove lignin, hemicellulose and other extractives^24,25^ was carried out for 2 hours at 75 °C, followed by 15 minutes in an ultrasonic bath at room temperature in the same bleach solution. Samples were then washed several times with MilliQ water to reach pH 5 and dried in an oven at 80 °C.

#### 2ChlorOx^3^

Two oxidative steps were applied. The first was performed in an alkaline environment at room temperature with NaClO and NaOH for 2 hours, followed by an acidic wash with HCl, and a second oxidative reaction with NaClO_2_ and HCl started at 70 °C for another 2 hours. These steps were then repeated. Following this procedure, samples were washed to neutrality and dried in an oven at 80 °C. This is in contrast to Gillespie's procedure,^3^ where the final product was freeze-dried.

After each step, samples were rinsed thoroughly with MilliQ water until the required pH was reached: ≤pH 10 before acid steps and ≥pH 4 before alkaline steps. To help solid–liquid separation samples were centrifuged and decanted. The weight of the initial dried sample and the weight of the dried final product were considered to calculate the cellulose yield % of each pretreated sample.

### Graphitisation and ^14^C dating

At the Ionplus laboratory, 2.5–3.0 mg dried cellulose was weighed into aluminium cups, compressed and then combusted in an elemental analyser coupled to an AGE 3. The resulting CO_2_ was converted into graphite in the reactors using H_2_ and 3.5 mg iron powder as a catalyst. Graphite was pressed into a target (sample holders) ready for AMS analyses at the ETH laboratory in Zurich. Samples of phthalic acid anhydride (chemical blank) and oxalic acid II (radiocarbon standard), kindly provided by the Ionplus laboratory, were graphitised and measured together with our samples in the ETH MICADAS (Mini Radiocarbon Dating System).^28^

## Results and discussion

**Fig. 1:**
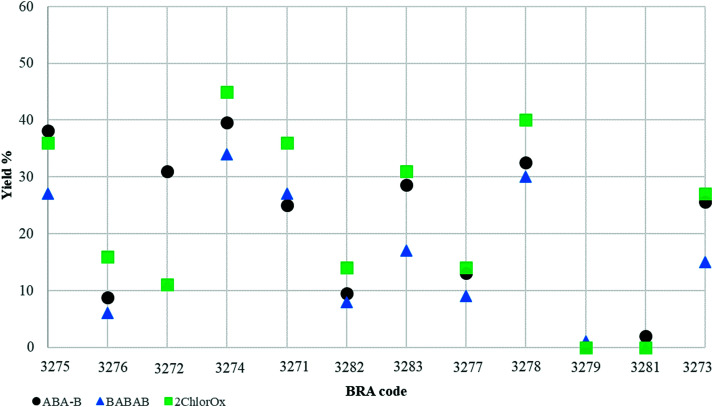
Comparison of sample cellulose yields (%) following pretreatment with the different methods (2ChlorOx, ABA-B, BABAB). Samples are ordered by age (from youngest to oldest).

The final cellulose yield of all pretreated samples is shown in Fig. 1. For two thirds of the triple tests, ABA-B and 2ChloOx resulted in a similar yield (within 5%). In contrast, for most samples, BABAB reduced the sample mass to lower than 35% of the initial weight. In general, all the pretreatment methods used in this study resulted in a substantial loss of the initial sample mass as only cellulose was targeted, which constitutes 40–44% of the dry weight of both soft- and hardwood trees^25^ and it is tightly connected to the state of wood preservation. 2ChlorOx was the most conservative among the considered protocols, together with ABA-B. Nevertheless, 50% of samples had a final yield between 20 and 40%, and the other half between 0 and 20%. In the BABAB procedure, in addition to the final bleaching stage, samples were first soaked in an alkaline bath of sodium hydroxide to dissolve humic acid and the polymeric molecules constituting lignin. The dissolution of lignin leads to a significant mass loss, as highlighted in Table 2: after the alkaline bath, 75% of our samples lost more than 30% of their initial weight. It is interesting to note the different behaviours of the background samples (BRA-3273, BRA-3279, BRA-3281) that show a significant loss of mass of 73% for BRA-3281, 50% for BRA-3279 and 46% for BRA-3273. At the end of the BABAB procedure, only BRA-3273 yielded sufficient cellulose to be radiocarbon dated. It is obvious that the pretreatment protocol adopted plays an important role in the isolation of cellulose and the quantity of datable material obtained. However, it is also true that the state of preservation, which is an intrinsic property of the sample, has to be taken into consideration when choosing the pretreatment method since it defines the available amount of cellulose. Samples BRA-3276, BRA-3279 and BRA-3281 are examples of this: for the last two samples (both background samples) the state of preservation was very low, and we could only extract 0–3% cellulose, which was not a sufficient amount to be ^14^C dated. Similarly, from the relatively young Holocene sample BRA-3276 only a small cellulose yield of <20% was obtained after the 2ChlorOx procedure, and <10% after the ABA-B and BABAB procedures. In contrast to the background samples, BRA-3276 is a relatively young (Holocene) pine wood which is poorly preserved. In general, we observed very different cellulose yields in both young (Holocene) and old (Upper Pleistocene) samples. There was no clear trend observable with burial time. Preservation depends on a combination of site-specific bedding conditions, burial time, and the durability of wood of different tree-species. In this respect, species such as oak, elm, pine and larch form heartwood in the inner part of the stem which contains phenolic compounds such as tannins, or other substances (*i.e.* resins), which make it much more resistant to decay than other types of wood, *i.e.* ash.^29,30^ The absence of residual cellulose from the background sample BRA-3279 may be due to a species-specific low resistance of ash, as a non-heartwood. Regarding the influence of burial conditions on the preservation of the trunk, we observe a similar situation between our sites, which enables good preservation of wood, even though the preservation status of individual trees from the different sites depends on the combination of all preservation factors. The subfossil trees from Hungary were found in Interglacial fluvial gravels which were part of the aquifer of the Danube River valley.^15^ As the trees were submerged in groundwater for most of the glacial period, they were predominantly exposed to anoxic bacterial decay. Trees from Furadouro were remnants of a pine forest in a lagoon close to the sea between 29 100–20 700 years BP, when the Atlantic ocean was below the present level.^12^ The site was covered by fluvial sandy sediments during the Pleniglacial phase of the last ice age. Due to sea-level rise in the post-glacial period, the trees were submerged after that time, which explains the low level of bacterial destruction (*i.e.* good cellulose preservation). The larch trees from Illegio^17^ (BRA-3277) and Revine^13^ (BRA-3274) and the pine from Cairo Montenotte (BRA-3278) were found in glacio-fluvial and colluvial sediments which kept the wood permanently under anoxic conditions and lead to remarkably good cellulose preservation, particularly at Revine and Cairo Montenotte. In comparison, the lower level of preservation at Illegio (BRA-3277) may be the result of local bedding conditions at that site. For the Holocene trees (BRA-3275; BRA-3276; BRA-3272) found in fluvial sediments,^18,19^ the permanently water-saturated conditions below the groundwater table was the crucial factor for high levels of preservation. The variation in preservation may be due to the local situation. The good preservation of the oak (BRA-3275) is due to the natural durability of this species due to the high content of tannins in oak heartwood.^30^

**Table 2:** Mass loss (%) for each sample after a 4% NaOH overnight bath at 75 °C in the BABAB procedure

Bologna lab code BRA-	Mass loss (%)
BRA-3275	39
BRA-3276	32
BRA-3272	30
BRA-3274	22
BRA-3271	51
BRA-3282	58
BRA-3283	27
BRA-3277	41
BRA-3278	39
BRA-3279	50
BRA-3281	73
BRA-3273	46

**Fig. 2:**
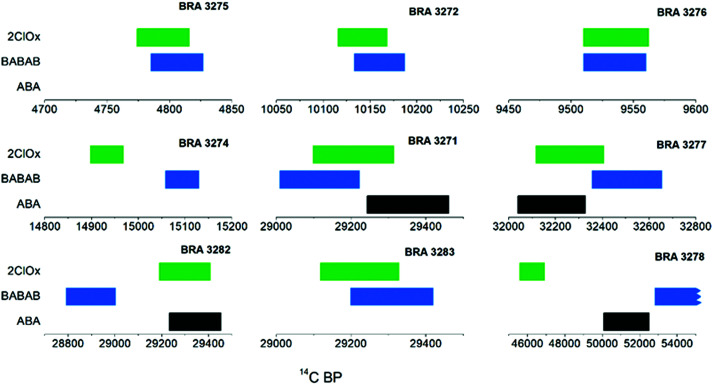
Comparison of ^14^C age ranges (1*σ*) of the pretreatment methods. 7 out of 9 samples (background sample BRA-3273 excluded) are within 2*σ* error range. BRA-3278 and BRA-3282 show the most significant differences.

The efficiency of contamination removal was evaluated through the ^14^C dating shown in Table 3 and Fig. 2. For the three dendro-dated samples (top row of Fig. 2) the calibrated ranges of the ^14^C ages cover the known calendar date. The measured ages of the sample pairs/triplets do not show significant variations. The results of the different pretreatment methods fall within the 2*σ* error range for 7 out of the 9 samples dated (background sample BRA-3273 excluded). The most significant difference is observed for sample BRA-3278: the ABA-B and BABAB extracts date to the background age level, whereas the 2ChlorOx extract is significantly younger. For BRA-3282, the BABAB extract is 2.7*σ* younger than the mean of the ABA-B and 2ChlorOx extracts. For the background sample BRA-3273, the F ^14^C (Fraction Modern, defined as the ratio of ^14^C specific activity of the sample and of the radiocarbon standard OxII, normalised to δ^13^C, as described by Stenström *et al.*^31^) values of the ABA-B and BABAB extracts (shown in Table 4) are essentially identical (0.00265), whereas the 2ChlorOx extract is higher (0.00290) by 2*σ* compared to the other two methods. It is also interesting to compare the age results obtained for sample BRA-3271 which was previously speed-dated (*i.e.* AMS dating of CO_2_ gas of untreated sample)^32^ and dated again in this study after the application of different pretreatment procedures. The result obtained from the untreated sample is younger by 2200 ^14^C years than the ages obtained from cellulose after pretreatment in this study. Contamination by 0.9% modern carbon in the untreated sample would account for this discrepancy.

**Table 3:** Carbon amount (%) following combustion and AMS ^14^C results of samples pretreated following the ABA-B, BABAB and 2ChlorOx protocols

Bologna lab code BRA-	Method	Carbon content (%)	F^14^C	±1*σ*	Age (^14^C BP)	±1*σ*
3275	BABAB	44.0	0.54978	0.00145	4806	21
	2ChlorOx	34.9	0.55050	0.00141	4795	21
3276	BABAB	42.4	0.30513	0.00097	9535	25
	2ChlorOx	40.3	0.30510	0.00097	9536	26
3272	BABAB	44.7	0.28228	0.00094	10 160	27
	2ChlorOx	44.2	0.28292	0.00093	10 142	26
3274	BABAB	42.2	0.15275	0.00068	15 093	36
	2ChlorOx	40.9	0.15584	0.00068	14 932	35
3271	ABA-B	42.4	0.02589	0.00035	29 352	109
	BABAB	41.6	0.02666	0.00035	29 115	107
	2ChlorOx	44.7	0.02636	0.00035	29 207	108
3282	ABA-B	43.5	0.02592	0.00035	29 343	110
	BABAB	40.4	0.02740	0.00036	28 897	106
	2ChlorOx	47.0	0.02606	0.00035	29 297	109
3283	BABAB	45.3	0.02603	0.00036	29 309	110
	2ChlorOx	51.4	0.02631	0.00036	29 223	105
3277	ABA-B	39.9	0.01820	0.00033	32 183	144
	BABAB	40.8	0.01748	0.00032	32 505	149
	2ChlorOx	31.6	0.01802	0.00032	32 261	145
3278	ABA-B	42.2	0.00169	0.00025	51 285	1 207
	BABAB	41.3	0.00112	0.00025	54 613	1 807
	2ChlorOx	43.2	0.00316	0.00026	46 254	661

**Table 4:** Background F^14^C values measured in this study

Bologna lab code BRA-	Method	F^14^C	±1*σ*
3273	ABA-B	0.00264	0.00008
	BABAB	0.00265	0.00009
	2ChlorOx	0.00290	0.00009

## Conclusions

The extraction and dating of cellulose from glacial sub-fossil wood are a key tool for improving the resolution of the radiocarbon calibration curve beyond the Holocene period. Cellulose extraction is fundamental to ensure correct dating. We aimed to determine the most effective extraction procedure on the basis of cellulose yield and ^14^C age, particularly focussing on trees grown in the glacial period (older than 14 226 years cal BP). In light of the obtained results, the ABA-B method was sufficiently conservative in term of cellulose yield and efficiently removed contamination even from samples older than 30 000 years cal BP. The 2ChlorOx method led to almost the same final yield of cellulose compared to ABA-B, but appeared slightly less capable of removing young contamination in very old and background samples. The BABAB method is the harshest pretreatment method to apply in the case of poorly preserved trees, and it led to the highest loss of material during pretreatment but the results were the most reliable. Therefore, the pretreatment procedure must be chosen according to the preservation state of the wood sample. Based on this study, we have adopted BABAB as our standard pretreatment protocol since it is the most adequate method and guarantees high ^14^C-dating quality. From our data and other studies referred to here, we can confirm that ABA-B pretreatment protocol represents a suitable alternative, especially for smaller sample quantities and wood samples with lower preservation status.

## Conflicts of interest

There are no conflicts to declare.
